# Oral fructose intake does not improve exercise, visual, or cognitive performance during acute normobaric hypoxia in healthy humans

**DOI:** 10.3389/fnut.2023.1170873

**Published:** 2023-07-21

**Authors:** Titiaan E. Post, Jan Schmitz, Cayla Denney, Riccardo De Gioannis, Henning Weis, Dominik Pesta, Andreas Peter, Andreas L. Birkenfeld, Sven Haufe, Uwe Tegtbur, Petra Frings-Meuthen, Ann C. Ewald, Daniel Aeschbach, Jens Jordan

**Affiliations:** ^1^Institute of Aerospace Medicine, German Aerospace Center (DLR), Cologne, Germany; ^2^Centre for Human Drug Research (CHDR), Leiden, Netherlands; ^3^Department of Anesthesiology and Intensive Care Medicine, Faculty of Medicine and University Hospital of Cologne, University of Cologne, Cologne, Germany; ^4^Department III for Internal Medicine, Faculty of Medicine, Heart Center, University Hospital of Cologne, Cologne, Germany; ^5^Department of Nuclear Medicine, Faculty of Medicine and University Hospital Cologne, University of Cologne, Cologne, Germany; ^6^Center for Endocrinology, Diabetes and Preventive Medicine (CEDP), University Hospital Cologne, Cologne, Germany; ^7^Cologne Excellence Cluster on Cellular Stress Responses in Aging-Associated Diseases (CECAD), University of Cologne, Cologne, Germany; ^8^Department for Diagnostic Laboratory Medicine, Institute for Clinical Chemistry and Pathobiochemistry, University Hospital Tübingen, Tübingen, Germany; ^9^Institute for Diabetes Research and Metabolic Diseases of the Helmholtz Center Munich at the University of Tübingen, Tübingen, Germany; ^10^German Center for Diabetes Research (DZD), Neuherberg, Germany; ^11^Division of Diabetology, Endocrinology, and Nephrology, Department of Internal Medicine, Eberhard Karls University Tübingen, Tübingen, Germany; ^12^Clinic for Rehabilitation and Sports Medicine, Hannover Medical School, Hannover, Germany; ^13^Institute of Experimental Epileptology and Cognition Research, University of Bonn Medical Center, Bonn, Germany; ^14^Medical Faculty, University of Cologne, Cologne, Germany

**Keywords:** normobaric hypoxia, fructose, exercise performance, visual performance, cognitive performance

## Abstract

**Introduction:**

The ability to metabolize fructose to bypass the glucose pathway in near-anaerobic conditions appears to contribute to the extreme hypoxia tolerance of the naked-mole rats. Therefore, we hypothesized that exogenous fructose could improve endurance capacity and cognitive performance in humans exposed to hypoxia.

**Methods:**

In a randomized, double-blind, crossover study, 26 healthy adults (9 women, 17 men; 28.8 ± 8.1 (SD) years) ingested 75 g fructose, 82.5 g glucose, or placebo during acute hypoxia exposure (13% oxygen in a normobaric hypoxia chamber, corresponding to oxygen partial pressure at altitude of ~3,800 m) on separate days. We measured exercise duration, heart rate, SpO_2_, blood gasses, and perceived exertion during a 30-min incremental load test followed by Farnsworth-Munsell 100 Hue (FM-100) color vision testing and the unstable tracking task (UTT) to probe eye-hand coordination performance.

**Results:**

Exercise duration in hypoxia was 21.13 ± 0.29 (SEM) min on fructose, 21.35 ± 0.29 min on glucose, and 21.35 ± 0.29 min on placebo (*p* = 0.86). Heart rate responses and perceived exertion did not differ between treatments. Total error score (TES) during the FM-100 was 47.1 ± 8.0 on fructose, 45.6 ± 7.6 on glucose and 53.3 ± 9.6 on placebo (*p* = 0.35) and root mean square error (RMSE) during the UTT was 15.1 ± 1.0, 15.1 ± 1.0 and 15.3 ± 0.9 (*p* = 0.87).

**Discussion:**

We conclude that oral fructose intake in non-acclimatized healthy humans does not acutely improve exercise performance and cognitive performance during moderate hypoxia. Thus, hypoxia tolerance in naked mole-rats resulting from oxygen-conserving fructose utilization, cannot be easily reproduced in humans.

## 1. Introduction

African naked mole-rats are unique animals which can withstand pain, cancer and even survive up to 18 min of anoxia without complications (1–3). Recent studies delineated the metabolic mechanism that explains how these mammals survive in habitats characterized by deep, crowded burrows with very low oxygen and high carbon dioxide levels (1, 4–6). One of the mechanisms enabling naked mole-rats to tolerate extreme hypoxia appears to be the ability to obtain energy from fructose during oxygen deprivation, bypassing the usual glucose pathway that requires oxygen. Under anaerobic conditions, glycolysis is blocked by feedback inhibition of phosphofructokinase via low pH, citrate, and allosteric binding of adenosine triphosphate. Fructose is metabolized through a pathway that bypasses the metabolic block at phosphofructokinase supporting viability. The metabolic rewiring of glycolysis avoids the usual lethal effects of oxygen deprivation, a mechanism that could be utilized to minimize hypoxic damage in human disease (1, 7). For example, such rewiring could be therapeutically exploited in patients with conditions resulting from systemic or organ-specific oxygen deprivation as in stroke and myocardial infarction (8, 9). Recent evidence suggests that hypoxia-inducible factor 1α (HIF-1α) impacts fructose metabolism in the context of cardiac pathologic stress-induced hypertrophic growth (10). Therefore, we tested the hypothesis that exogenous fructose improves acute hypoxia tolerance in humans. Given the limited evidence regarding human fructose metabolism, we first assessed the systemic availability and metabolic response following oral fructose loading. Then, we determined whether fructose ingestion acutely improves endurance capacity and visual and cognitive performance in humans exposed to acute hypoxia in a randomized, double-blind, placebo-controlled, and crossover study.

## 2. Methods

### 2.1. Participants

The study comprised two substudies. In the first substudy, we assessed systemic availability and metabolic effects of oral fructose. Because the main goal of this study was to determine to what extent oral fructose reaches the systemic circulation, the experiment was conducted in normoxic conditions. In the second substudy, we compared influences of placebo, fructose, and glucose ingestion on physical, visual and cognitive performance in normobaric hypoxia. In both substudies, we included healthy persons following screening comprising a physical exam and routine laboratory testing. Men and women aged 18–45 years with a body mass index between 18 and 25 kg/m^2^ were eligible. We excluded participants with fructose malabsorption determined by fructose malabsorption testing (Gastro+^™^ Gastrolyzer^®^, Bedfont Scientific, UK). In substudy 2, additional inclusion criteria were being recreationally active defined as biking >2 h or running >1 h per week and no color blindness (24-plates Ishihara). We obtained written informed consent prior to the start of the study. Both substudies were approved by the North Rhine Medical Association (Ärztekammer Nordrhein) and prospectively registered at the German Clinical Trials Register (DRKS, registration numbers: DRKS00028599 and DRKS00028644). We conducted all procedures according to the Declaration of Helsinki. Study participants were compensated for their participation. We performed medical screening and all experiments at the German Aerospace Center (DLR) in Cologne, Germany.

### 2.2. Substudy 1—systemic fructose availability and metabolic actions

We included 8 healthy participants (4 women, 4 men; mean age ± SD: 25.5 ± 3.6 y). Participants abstained from intense physical activity for 1 day prior to the study day and maintained a food diary for 3 days prior to the study day. After a 10 h fast, they reported to the laboratory. A venous catheter was placed in the forearm and participants remained in a supine position for 10 min before being placed under the respiratory hood and initiation of the assessment. Following baseline measurements, participants ingested 75 g fructose dissolved in 300 mL water. We obtained venous blood for fructose, glucose, insulin, free fatty acids (FFA), and triglyceride measurements before and 15, 30, 45, 90, 120, 150, 180 min following fructose ingestion. Plasma fructose concentrations were determined using a manual colorimetric assay (Abnova). Glucose and triglyceride levels were assessed using the ADVIA XPT clinical chemical system (Siemens Healthineers, Eschborn, Germany). Serum insulin was determined using an immunoassay on an ADVIA Centaur XPT system (Siemens Healthineers, Eschborn, Germany). Plasma concentrations of total FFA were measured with an enzymatic method (WAKO Chemicals, Neuss, Germany) on the latter instrument. We assessed resting metabolic rate and substrate oxidation (using the Weir equation) through indirect calorimetry (Quark RMR, COSMED) (11). After a 30-min baseline, changes in substrate oxidation were assessed in three different intervals 5–45 min, 65–80 min, and 95–115 min after fructose ingestion.

### 2.3. Substudy 2—randomized, crossover comparison of placebo, glucose, and fructose in hypoxia

**Figure 1:**
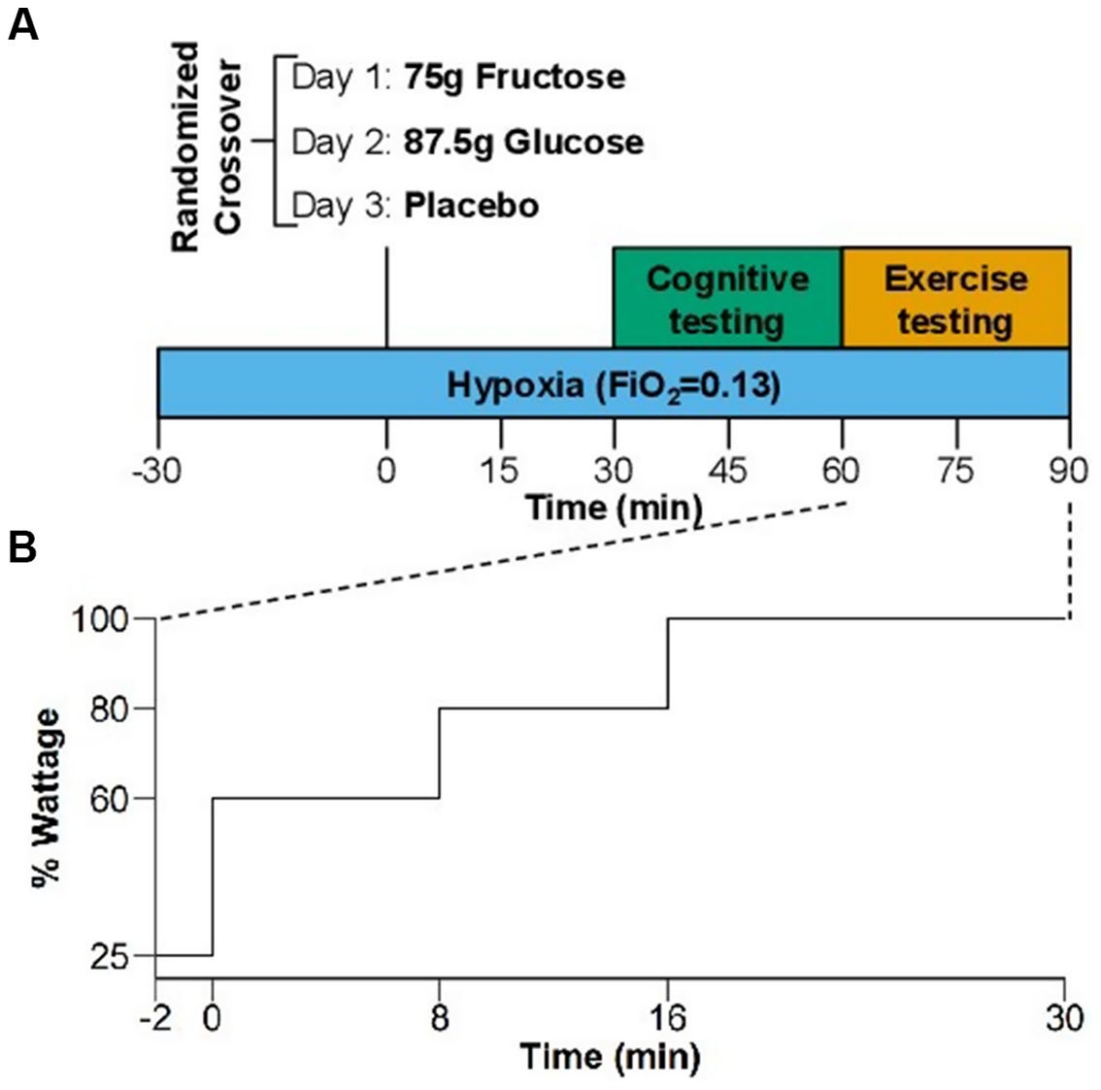
**(A)** Study design of the randomized, crossover comparison of effects of placebo, glucose, and fructose in hypoxia. Blue bar indicates the 2 h hypoxia exposure (FiO_2_ = 0.13). Green panel shows the visual and cognitive test and orange panel the exercise testing. **(B)** Exercise protocol of the increasing load test in % of the estimated maximum exercise capacity using normative values for maximal workload (see Methods section).

We included 26 healthy participants (9 women, 17 men; mean age ± SD: 28.8 ± 8.1 y). The substudy comprised a familiarization period followed by a randomized, double-blind, crossover comparison of placebo, glucose, and fructose in a normobaric hypoxia chamber (Figure 1A). In the familiarization period, participants practiced exercise, visual and cognitive tasks in normobaric hypoxia (FiO_2_ = 13.0%, equivalent to a simulated altitude of 3,800 m) on two separate days.

Six to nineteen days following the familiarization period, we randomized participants to one of six groups (fructose-glucose-placebo, fructose-placebo-glucose, glucose-fructose-placebo, glucose-placebo-fructose, placebo-fructose-glucose and placebo-glucose-fructose). Thus, we examined each participant on three separate study days with a three-day recovery between visits under identical atmosphere but following ingestion of 75 g fructose (Euro OTC Pharma GmbH, Bönen, Germany), 82.5 g glucose (isocaloric to fructose) (Caelo, Hilden, Germany), or placebo (non-caloric, 200 mg saccharin/Buxtrade, Buxtehude, Germany) dissolved in 300 mL water.

One week before each study day, participants abstained from recreational drugs, caffeine, alcohol, and nicotine, which was verified by urine testing on study days. Subjects also abstained from intense physical activity for 24 h before study days. To limit influences of sleep deprivation and circadian misalignment on our measurements, participants maintained regular bedtimes and rise times (time in bed: 8 h) during the 3 days prior to each study day, confirmed with wrist-actigraphy (Actiwatch-L; Philips/Respironics). In each participant, all three study days commenced at the same time, but varied among participants between 8 a.m. and noon. Participants arrived after 10 h overnight fasting and entered the hypoxia module. We placed a venous catheter in the forearm and participants remained in a seated position before and between exercise and cognitive testing. After 30 min of hypoxia exposure participants ingested fructose, glucose, or placebo within 1 min. Thirty minutes later, participants performed the visual and cognitive tasks, which took approximately 30 min to complete and that were followed by the incremental load exercise test. Thus, exercise testing coincided with expected systemic fructose and glucose peak concentrations following oral ingestion. During the experiment in hypoxia, participants were limited to drink only water.

### 2.4. Normobaric hypoxia

We achieved hypoxia by nitrogen dilution through the air conditioning system in the atmospheric self-sustaining hypoxia chamber under normobaric conditions (1,013 hPa). Nitrogen was supplied by an external tank. Participants were exposed to normobaric hypoxia (FiO_2_ = 0.13) for 2 h without pre-acclimatization. The normobaric hypoxia condition with an oxygen fraction of 13% corresponds to the partial oxygen pressure present at an altitude of approximately 3,800 m. We chose this altitude and exposure time so that the selected visual, cognitive and exercise tests were sensitive to measure a performance impairment, while simultaneously being performed safely and without symptoms of high mountain sickness developed (12–15).

### 2.5. Color vision and cognitive testing

We tested color vision with the FarnsworthMunsell 100-Hue (FM-100) test (Luneau, Paris). Test illuminance, color temperature of the light source, procedure, and data processing adhered to the original Farnsworth instructions (16). The test consists of 4 sub-sectors: sector 1 (caps 76–12), sector 2 (caps 13–33), sector 3 (caps 34–54), and sector 4 (caps 55–75). The total error score (TES, sum of scores for each four sectors), total number of errors in each sector and in the red-green (caps 13–33 and 55–75) and blue-yellow axis (caps 1–12, 34–54, and 76–85) were calculated (17). The unstable tracking task (UTT) is an eye-hand coordination test, in which a horizontally and continuously moving cursor has to be centered within a marked target located in the middle of the screen by moving a lever to the left or right with the dominant hand. The task duration is 3 min according to the recommendation of the AGARD Handbook and was shown to be sensitive to the stressors. Performance was measured as a root mean square error (RMSE) of the distance of the cursor to the center (18–20).

### 2.6. Exercise testing

Exercise testing was done on an ergometer bike (COSMED). We estimated participants’ exercise capacity using normative values for maximal workload of the average population (in watts) normalized for age, sex, and body weight (21). Our population was more athletic but performed exercise in hypoxia, so we tried three different protocols prior to the start of the study (40–60–80%, 50–60–90% and 60–80–100%, for 8–8–14 min, respectively). The latter proved to be the most suitable for our population. The exercise protocol consisted of a 2-min warm-up at 25% of the estimated maximal exercise capacity followed by a 30-min incremental load test with 8 min at 60%, 8 min at 80%, and up to 14 min at 100% of estimated maximal exercise capacity (Figure 1B). We instructed participants to maintain a cadence between 70 and 90 rpm and to exercise as long as tolerated in case they could not complete the 30-min test. We measured heart rate (Philips ECG IntelliVue MX40) and blood oxygenation (finger pulse oximeter) at 1-min intervals throughout the exercise test. Moreover, we assessed venous blood gasses (ABL90 FLEX analyzer, Radiometer Medicals ApS, Denmark) and perceived exertion using the BORG scale before and during the bike test at the end of each incremental step (the last measurement was taken when the test was stopped at voluntary exhaustion).

### 2.7. Study endpoints and sample size justification

Substudy 1 was an exploratory investigation with the primary goal to test whether oral fructose ingestion increases systemic fructose availability. We did not conduct a formal sample size estimation for this substudy. In substudy 2, the primary endpoint was the difference in exercise performance between fructose, glucose and placebo, measured as bike duration till voluntary exhaustion (min) and heart rate (bpm) during the increasing load exercise test. Secondary outcomes of the exercise tests were blood oxygen content, blood lactate concentrations and BORG score measured at the end of each incremental step. TES during the FM-100 and RMSE during the UTT were also considered secondary outcomes. A statistical power analysis (GPower 3.1 software) was performed for sample size estimation, assuming a moderate effect of fructose ingestion on bike duration and heart rate during an increasing load exercise test, according to Cohen (1988) (22) with 0.296 effect size. We chose a more cautious effect size because there was no clear evidence as to whether humans benefit from exogenous fructose under hypoxic conditions. We used alpha = 0.05 and power of 0.80, applied in three groups (fructose, glucose and placebo) analyzing the results through an analysis of variance (ANOVA) with repeated measures and post-hoc comparison test for a comparative analysis within groups. The projected sample size needed with this effect size was approximately *n* = 20 for a crossover design.

### 2.8. Statistical analysis

Statistical analysis was performed using RStudio software (version 2022.07.1). If not otherwise indicated, data of substudy 1 are reported as mean ± SD and substudy 2 mean ± SEM. Kaplan–Meier (KM) curve was used to analyze and compare the survival differences between the three treatment conditions during the increasing load test. The other outcomes were analyzed using a linear mixed model with time and condition as fixed factor, and participant as random factor.

## 3. Results

### 3.1. Substudy 1—systemic fructose availability and metabolic actions

**Figure 2:**
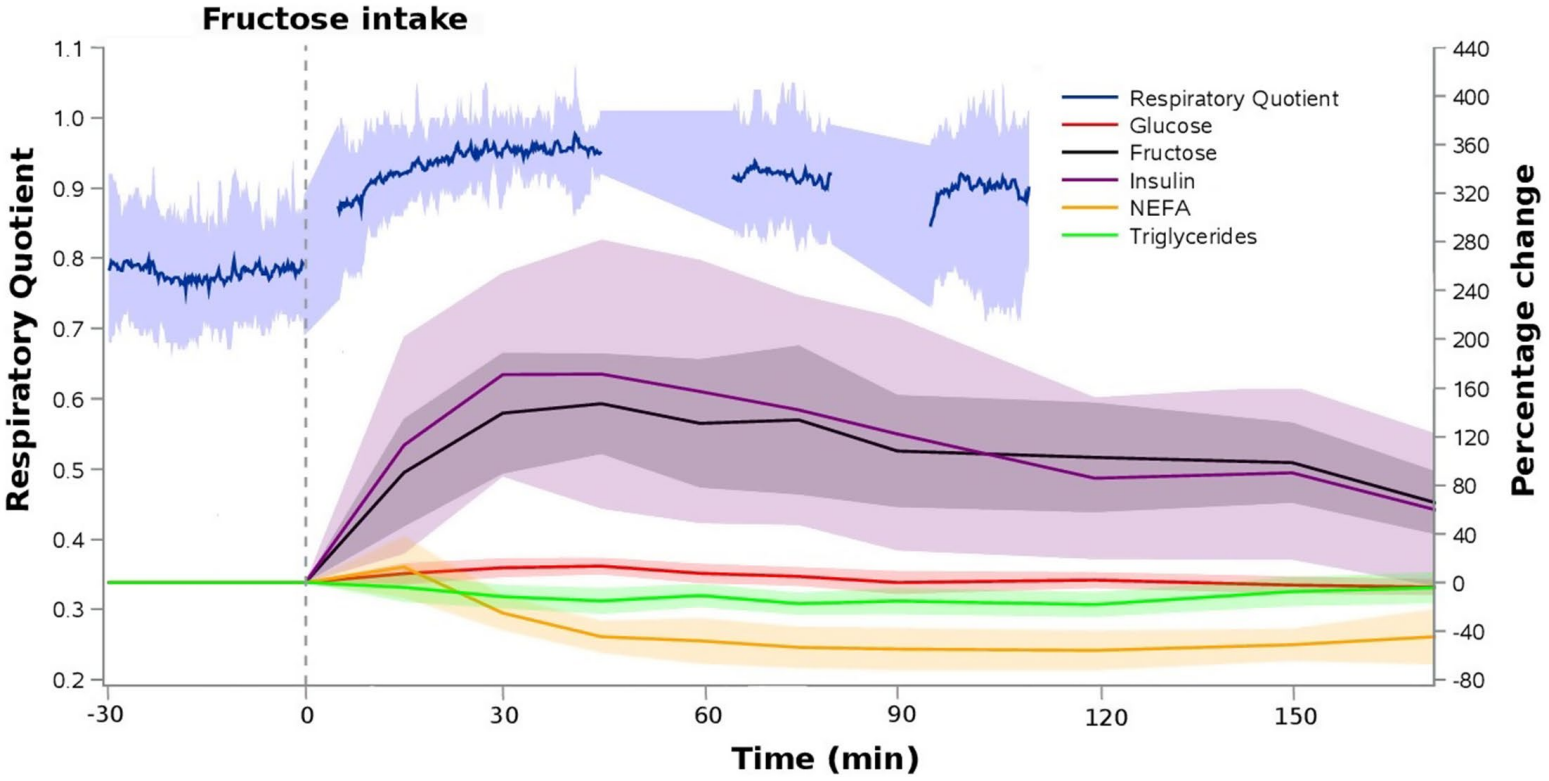
Relative time profiles of blood metabolites and respiratory quotient following 75 g oral fructose ingestion. Data represents means ± SD (*N* = 8).

Figure 2 illustrates the time course of fructose, glucose, insulin, non-esterified fatty acids, triglycerides and the respiratory quotient following fructose ingestion. Mean plasma fructose concentration was 61.4 ± 7.4 μg/mL at baseline and rapidly increased to 145.5 ± 27.9 μg/mL and 150.4 ± 23.2 μg/mL 30 and 45 min after fructose ingestion, respectively. Three hours after fructose ingestion, plasma fructose concentration was 93.0 ± 9.5 μg/mL. Insulin increased from 53.1 ± 22.2 pmoL/L at baseline to 132.7 ± 46.2 pmoL/L at 30 min and 128.2 ± 44.4 pmoL/L at 45 min after fructose ingestion. Three hours after fructose ingestion, insulin concentration was 71.7 ± 26.5 pmoL/L. Triglycerides levels slightly decreased from 74.9 ± 35.6 mg/dL at baseline to 60.1 ± 27.6 mg/dL 120 min after fructose ingestion. Non-esterified fatty acids levels decreased from 697.9 ± 242.2 μmol/g at baseline to 297.4 ± 129.9 μmol/g 120 min after fructose ingestion. Glucose concentration was 88.5 ± 5.7 mg/dL at baseline and 100.2 ± 4.5 mg/dL 45 min after fructose ingestion. Mean fasting respiratory quotient was 0.79 ± 0.09 at baseline and increased to 0.96 ± 0.07 30 min and 0.96 ± 0.07 45 min after fructose ingestion consistent with a relative shift from fatty acid to carbohydrate oxidation.

### 3.2. Substudy 2—randomized, crossover comparison of placebo, glucose, and fructose in hypoxia

Four participants were excluded from the study due to missing data on one of the three visits (two participants developed a COVID infection and two vasovagal syncope related to multiple venipuncture attempts on visit one or two and we decided to discontinue the study). Two subjects developed vasovagal syncope during the exercise test on their last visit and were only excluded for the exercise analysis. This resulted in 22 complete datasets for the cognitive performance analysis and 20 complete datasets for the exercise performance analysis.

**Figure 3:**
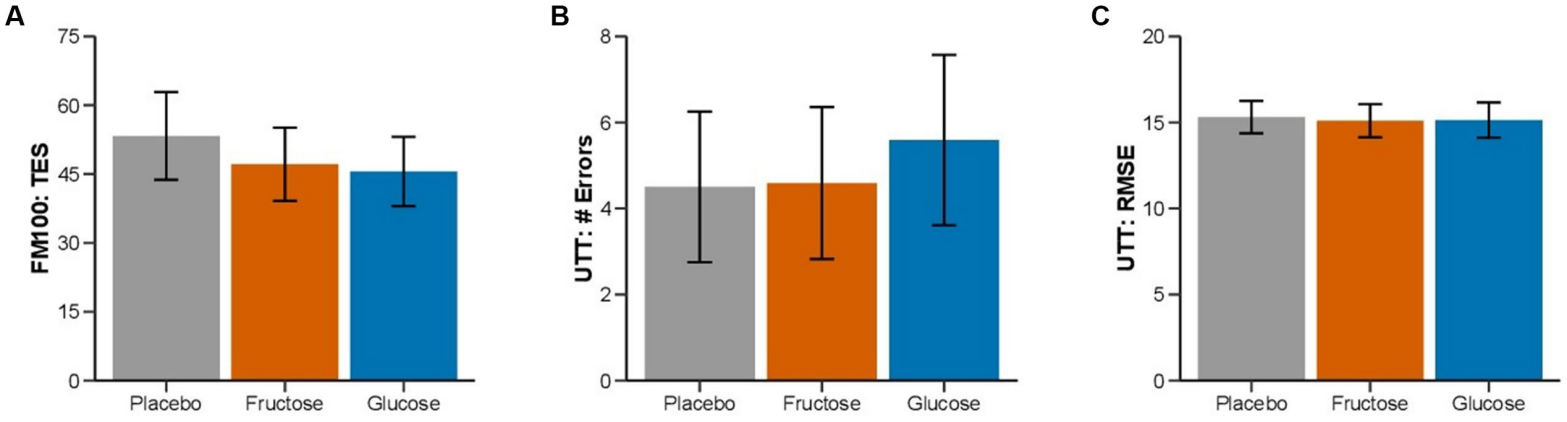
Effect of oral fructose, glucose or placebo intake on color vision and cognitive performance: Mean (SEM) of **(A)** total error score (FM-100), **(B)** lapses, and **(C)** tracking deviation (UTT) of the three treatment conditions.

**Figure 4:**
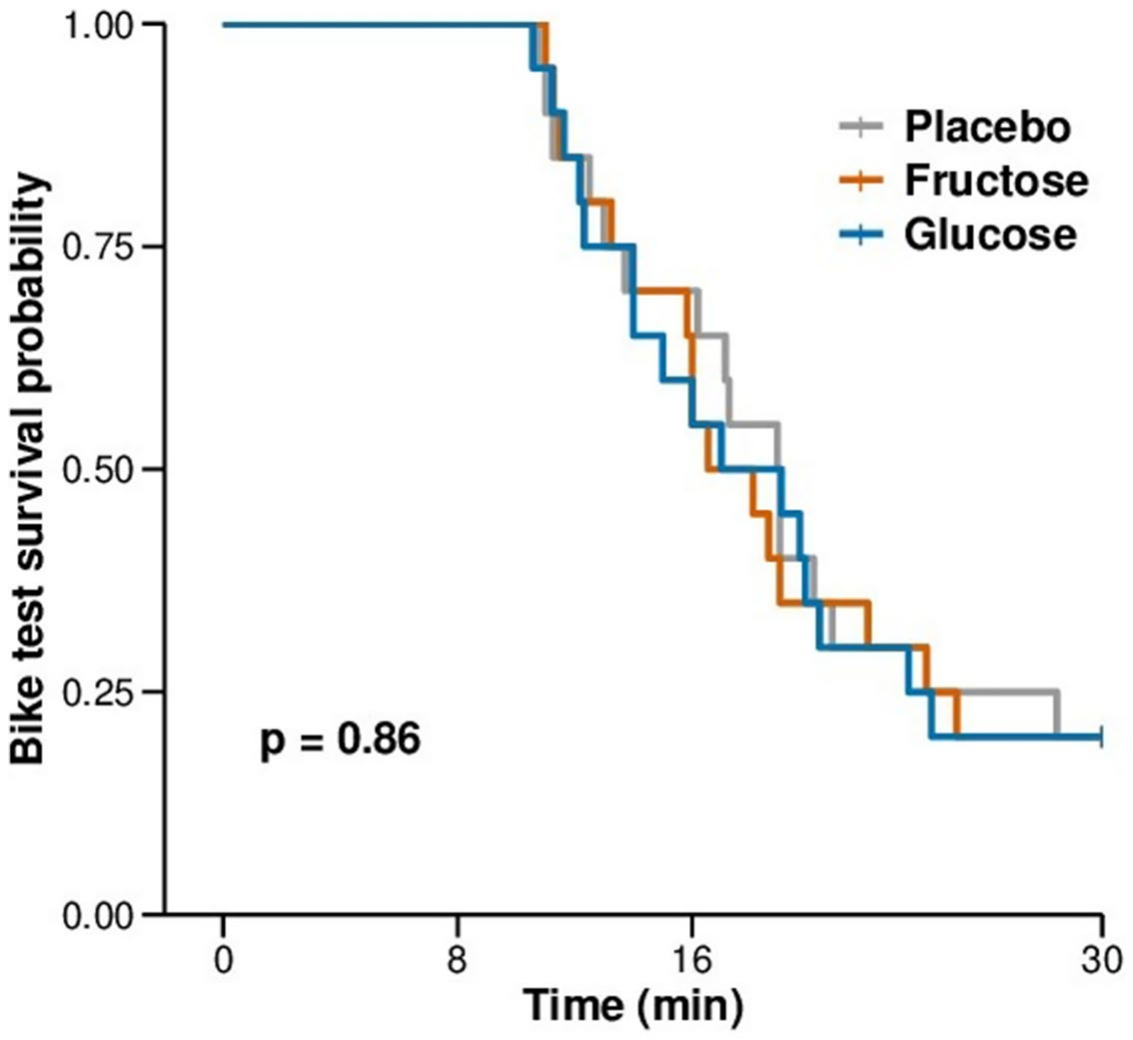
Kaplan-Meier plot of the duration of exercise to exhaustion in minutes during the exercise test in hypoxia.

**Figure 5:**
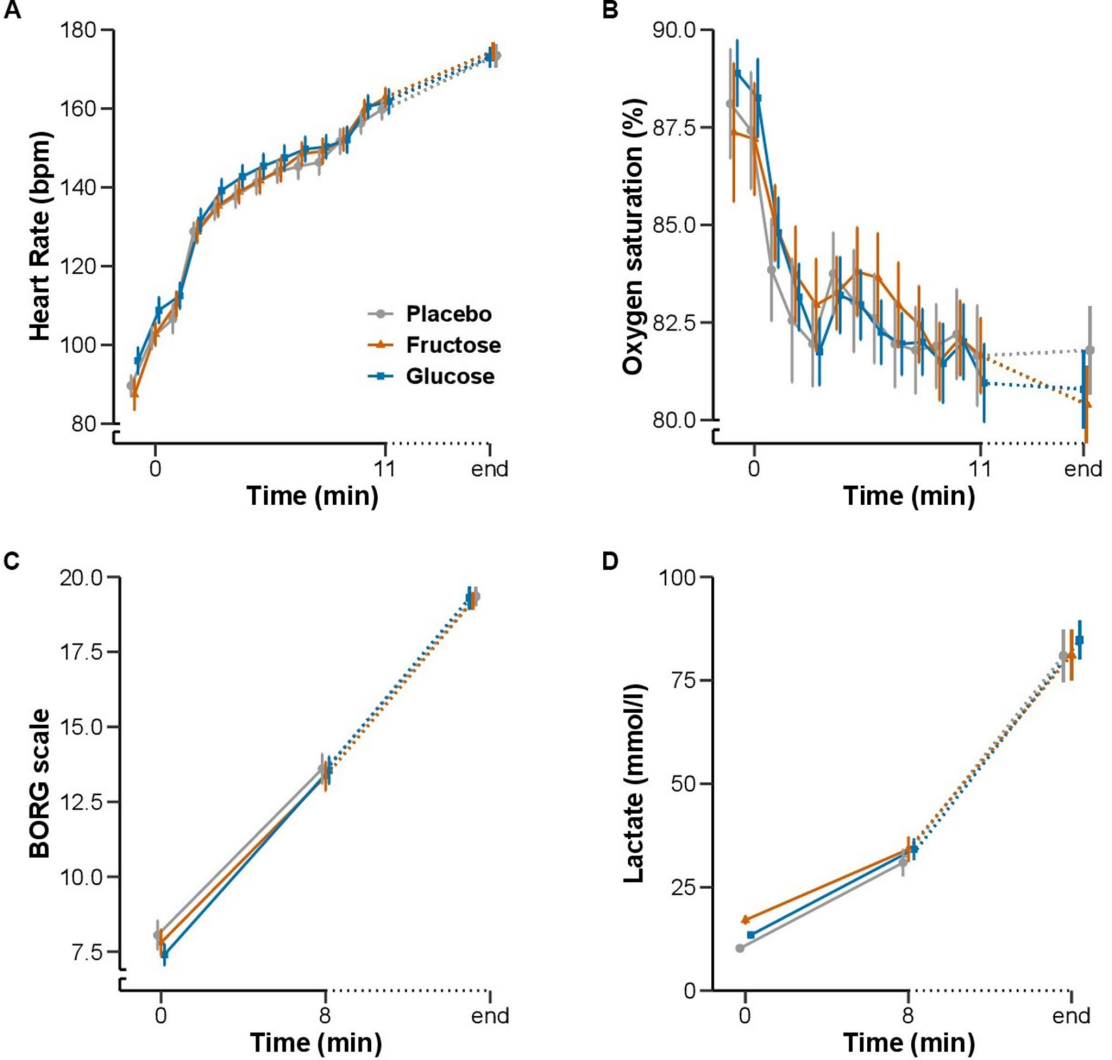
Effect of oral fructose, glucose or placebo intake on exercise performance during acute hypoxia: Mean (SEM) of **(A)** heart rate (bpm) and **(B)** oxygen saturation (%) in minute intervals from baseline to 11 min and at the end of the exercise test. **(C)** BORG scale and **(D)** blood lactate concentration (mmol/l) at baseline, 8 min and at the end of the exercise test.

None of the measurements of the FM-100 and UTT test showed significant differences between the three conditions (Figure 3). All participants completed 11 min of bike exercise before the first participants finished the test at voluntary exhaustion (after minute 3 at 80% of maximal workload). Four participants completed the entire 30-min period (Figure 4). We observed no differences between conditions regarding the time to exhaustion. At the start of the exercise test, 1.5 h into hypoxia exposure, heart rate was 89.7 ± 2.6 bpm on placebo, 87.4 ± 3.9 bpm on fructose, and 96 ± 3.4 bpm on glucose. At the end of the exercise test, heart rate was 159.8 ± 2.6 bpm on placebo, 162.8 ± 2.4 bpm on fructose, and 161.8 ± 3.1 bpm on glucose. At this time perceived exertion according to the BORG scale was 19.4 ± 0.3, 19.3 ± 0.2, and 19.4 ± 0.3 with placebo, fructose, and glucose, respectively (Figure 3). No significant differences between the three treatments were observed for heart rate, BORG scale and SpO_2_. Lactate and pO_2_increased and pCO_2_ decreased significantly during the exercise test but did not show significant differences between the three conditions (Figure 5).

## 4. Discussion

We showed here that acute ingestion of fructose increases its systemic availability in healthy humans, but does not augment physical, visual or cognitive performance during exposure to moderate hypoxia. Indeed, time to exhaustion, perceived exertion, heart rate, oxygen saturation, and blood gasses during physical exercise in hypoxia were virtually identical following ingestion of fructose, glucose, or placebo. Similarly, fructose ingestion had no effect on color vision or cognitive performance in hypoxia. Thus, the remarkable hypoxia tolerance in naked mole-rats, which is achieved in part through metabolic rewiring toward reduced oxygen demand by fructose utilization, cannot be reproduced in humans through fructose administration.

While fructose utilization plays a significant role, the hypoxia tolerance of naked mole rats is not solely reliant on this mechanism. These animals possess a range of additional coping mechanisms that collectively contribute to their remarkable survival in low-oxygen environments. These mechanisms include efficient oxygen utilization due to their lower metabolic rate, enhanced oxygen binding capacity through high hemoglobin-oxygen affinity, tolerance to high carbon dioxide levels, resistance to acidosis facilitated by improved buffering systems and efficient acid removal, as well as antioxidant defenses protecting brain cells from oxidative stress and reducing metabolic demands (1, 23–25). Together, these adaptations highlight the complex and multifaceted nature of their hypoxia tolerance. These observations also suggest that in humans metabolic rewiring toward fructose metabolism may not suffice to enhance performance under hypoxic conditions.

We determined established hypoxia-sensitive responses in a randomized, double-blind, placebo-controlled, and crossover fashion, which is a strength of our study. Performance during endurance exercise testing decreases substantially in hypoxia with concomitant increases in perceived exertion and heart rate, and reductions in oxygen saturation. A systematic review highlighted ergolytics effect of acute hypoxia exposure on time to exertion during physical exercise in relation to hypoxia severity and test duration (26). A level of 13% of oxygen exacerbates peripheral fatigue of limb locomotor muscles, which likely contributes to early termination of exercise (27). In our study, most participants did not complete the full exercise protocol leaving room for potential improvements on treatment. We chose visual and cognitive tests known to be sensitive to 13% O_2_. Both tests are sensitive at an altitude of 3,000 m, reflected in an increase in total number of errors in the FM-100, and an increase in tracking deviations in the UTT (12, 13). Reduced oxygen saturation during exercise in normobaric hypoxia (FiO_2_ = 0.135) contributes to exercise-induced cognitive fatigue (28). To exclude a possible interaction effect of hypoxia and exercise we therefore scheduled visual and cognitive testing to occur prior to the exercise test.

Fructose is rapidly cleared by the intestines and liver and is catabolized for energetic purposes, converted into glucose, and stored as glycogen, or converted into fatty acids and stored as triglycerides (29). Dietary fructose (75 g) acutely increases systemic serum fructose levels and elicits acute metabolic and endocrine responses in humans (30). Thus, fructose at a dose applied in our study in addition to resulting in significant increases in systemic fructose availability elicited changes in insulin release and carbohydrate metabolism. In previous studies, changes in systemic glucose following fructose ingestion were rather small (31). There may have been a small increase in glucose in our study. We speculate that the increase in circulating glucose may have been attenuated by increased glucose disposition as we studied relatively young and insulin sensitive persons. We selected a 75 g fructose dose for experiments under hypoxia because the dose is sufficient to increase systemic fructose availability (substudy 1), challenges the metabolic system [substudy 1 and (30)], surpasses typical dietary intake, is well tolerated, and could be reasonably applied in subsequent interventions studies in healthy persons or patients. Because fructose concentrations and associated metabolic changes reached a peaked approximately 30 min after fructose ingestion, we tested cognitive and visual performance in hypoxia in this period.

Because performance could be affected by a placebo effect, we decided to use saccharin as a placebo to mimic the sweetness of glucose and fructose. It is known that artificial sweeteners, including saccharin, can impact brain activity (32). However, no association has been found between the intake of artificial sweeteners in various forms and cognitive performance, as measured by an array of tests (33). Furthermore, several studies suggest that when compared to saccharin, glucose or sucrose (glucose-fructose) elicit more pronounced brain responses (34–36).

Compared with placebo and glucose ingestion, fructose ingestion was not associated with changes in hypoxia tolerance. Possibly, the negative findings of our study could be explained by fructose dosing, the level of hypoxia, or species differences between naked mole-rats and other mammals including humans. While we showed in our pilot study that orally ingested fructose reaches the systemic circulation, first pass metabolism may nevertheless have limited peripheral fructose availability (37, 38). Moreover, the administration of a single fructose dose may not have allowed sufficient time and doses to observe a metabolic shift in humans. Furthermore, high fructose concentrations could lead to substrate inhibition of its transporters and enzymes, limiting fructose absorption, metabolism, and utilization.

The naked mole-rat has developed the ability to use fructose to fuel vital organs such as the brain and heart under near-anaerobic conditions. This metabolic rewiring supplies metabolically active organs with transporters and enzymes that are required to metabolize fructose to lactate. Fructose metabolism relies on ketohexokinase enzymes and GLUT-5 transporters, which in most mammals studied so far appear to be present exclusively in the liver and kidney (39). GLUT-5 has been identified in smaller quantities in several adult human tissues, including the kidney, brain, muscle, and adipose tissue (40, 41). However, the specific physiological relevance of GLUT-5 in these tissues is currently unknown. In contrast, the naked mole-rat showed significant presence of these two key factors even in heart and brain tissue (1).

## 5. Limitations

No dietary questionnaire was conducted to determine whether the recruited subjects were accustomed to eating more or less fructose. Moreover, we excluded participants with fructose malabsorption based on a 25 g fructose dose. In a dose–response study of fructose absorption during a breath test in healthy subjects, 10% of individuals tested positive for malabsorption with a 25 g dose of fructose, while 80% tested positive with a 50 g dose, which could have confounded our study (42). Yet, we are confident that we exclude participants with more severe fructose malabsorption and we reached a substantial increase in systemic fructose availability with a 75 g fructose dose. Another potential limitation is that we conducted the study on systemic fructose availability following oral ingestion under normoxic conditions. We cannot exclude that hypoxia may have changed the response. Furthermore, in substudy 2 we estimated exercise capacity rather than determining it individually by VO_2_ max measurement at baseline. The rational was that we attempted to limit the number of study visits during a peak period of the COVID pandemic. Moreover, although FM-100 and UTT are known to be sensitive to hypoxia exposure, the tests may not suffice to detect subtle changes in visual or cognitive function. It is important to note that our study lacked baseline measurements in normoxia, which limits our ability to asses individual hypoxia sensitivity. Yet, acute fructose or glucose ingestion did not improve performance in normoxia (43). Although the sensitivity of the cognitive and visual test is supported by the literature, there are differences in the protocol used in those studies. Consequently, we cannot be certain that our protocol produces changes in cognitive and visual performance. Thus, our findings cannot be simply extrapolated to other populations. Whether administering a single fructose dose is sufficient to improve cognitive and physical performance in hypoxia is uncertain. In terms of hypoxia tolerance, multiple fructose doses ingested over several days may be more effective. We dare to speculate that multiple fructose doses could induce metabolic adaptation necessary for fructose metabolism. However, potential improvements in hypoxia tolerance have to be weighed against adverse effects on cardiovascular and metabolic risk with long-term exposure to increased dietary fructose. To achieve better results, a longer period of acclimatization to hypoxia through HIF-dependent expression of transporters and enzymes, as well as to fructose, would likely be necessary.

## 6. Conclusion

Oral intake of a single 75 g fructose dose in non-acclimatized healthy humans compared to glucose and placebo does not improve visual, cognitive or exercise performance during moderate normobaric hypoxia. While oral fructose ingestion may be ineffective in improving hypoxia tolerance in humans, the metabolic pathways could nevertheless have physiological and clinical relevance. It is tempting to speculate that fructose-metabolizing pathways could be upregulated to ameliorate consequences of systemic or localized hypoxia. A switch to fructolysis under hypoxic stress has been associated with heart failure, metabolic syndrome and malignant cancer. This hints at a potential link between the metabolic adaptation of the naked mole-rat to chronic hypoxia and its resistance to cancer. It is therefore important to understand whether, in humans, chronic adaptation to hypoxia is required to induce metabolic changes necessary to benefit from fructose. Future studies in humans are needed to rule out this question.

## Data availability statement

The raw data supporting the conclusions of this article will be made available by the authors, without undue reservation.

## Ethics statement

The studies involving human participants were reviewed and approved by North Rhine Medical Association (Ärztekammer Nordrhein). The patients/participants provided their written informed consent to participate in this study.

## Author contributions

TP, JS, RG, CD, PF-M, AE, SH, UT, DA, DP, and JJ designed the study. TP, JS, CD, PF-M, AE, and RG collected the data. JS, RG, HW, and JJ were the responsible physicians. TP, JS, and CD were responsible for the experimental conditions. TP, RG, PF-M, AE, and AP analyzed data. AB and AP measured blood concentration. DA and JJ provided critical insights into the data analyses and interpretation. TP wrote the original draft. All authors edited and reviewed the manuscript.

## Funding

The study was supported through funds from the Aeronautics and Space Programs of the German Aerospace Center.

## Conflict of interest

The authors declare that the research was conducted in the absence of any commercial or financial relationships that could be construed as a potential conflict of interest.

## Publisher’s note

All claims expressed in this article are solely those of the authors and do not necessarily represent those of their affiliated organizations, or those of the publisher, the editors and the reviewers. Any product that may be evaluated in this article, or claim that may be made by its manufacturer, is not guaranteed or endorsed by the publisher.
